# High bending strength at 1800 °C exceeding 1 GPa in TiB_2_-B_4_C composite

**DOI:** 10.1038/s41598-023-33135-w

**Published:** 2023-04-27

**Authors:** A. Kuncser, O. Vasylkiv, H. Borodianska, D. Demirskyi, P. Badica

**Affiliations:** 1 National Institute of Materials Physics, Street Atomistilor 405 A, 077125 Magurele, Romania; 2 National Institute for Materials Science, 1-2-1 Sengen, Tsukuba, Ibaraki 305-0047 Japan; 3 WPI-Advanced Institute for Materials Research (WPI-AIMR), Tohoku University, 2-1-1 Katahira, Aoba-ku, Sendai, 980-8577 Japan; 4 Department of Materials Science and Engineering, Tohoku University, 6-6-02 Aramaki Aza Aoba, Sendai, 980-8579 Japan

**Keywords:** Ceramics, Structural properties

## Abstract

High density (99.5%) ceramic composite composed of titanium boride and boron carbide (70/30 vol%) was obtained by spark plasma sintering and was tested by 3-point bending test in Ar atmosphere at 1800 °C. Bending strength was high, around 1.1 GPa. The strength–strain curve presents a peculiar shape composed of three regions where elastic and plastic deformations are active with a different weight. Based on transmission electron microscopy observations we propose a process of mechanical energy absorption driven by shear stress in the boron carbide crystals: stacking faults with (1-11) and (011) stacking planes and twins with (1-11) twinning plane rearrange into nano-twins with (10-1) twinning planes, orthogonal but equivalent to the initial ones. This rearrangement mechanism provides in the first instance a plastic signature, but further contributes strengthening.

## Introduction

Technological advancements in strategic domains such as nuclear energy, and aerospace industries are primarily related to the engineering of innovative advanced functional materials^1^. Such materials for extreme conditions should be able to withstand very high temperatures, possess high hardness, toughness, and ideally a good thermal, electrical conductivity, and chemical stability. All the above-mentioned features should occur simultaneously. Moreover, production of these materials should be cheap, fast, and scalable^2^.

Only few families of materials^3^ meet the particular, narrow set of above-mentioned requirements. Among them are refractory metals (e.g. W and Mo), oxides (ZrO_2_ and MgO), borides (TiB_2_ and TaB)^4^, carbides (TaC, ZrC, and TiC) or nitrides (TaN and HfN). In general, at room temperature metals are ductile and undergo plastic deformation, while ceramics are brittle, hard, and deform elastically. However, totally unexpected, a deformation behavior accompanied by unusual physical mechanisms of deformation may occur. For example, some ceramics such as tantalum carbide (TaC), hafnium boride (HfB_2_) and boron carbide (denoted BC) are able to accommodate at high temperatures plastic deformation similar to metals due to e.g. the dynamics of crystallographic defects^5–8^. Under mechanical load, the interplay between the intrinsic properties of the material (crystal chemistry and defects) and microstructure at nano and micro scale (grain size, distribution, and shape and grain boundaries) can promote novel physical mechanisms of energy relaxation. These mechanisms result in the peculiar profiles of the stress vs. strain curves. In addition, it is well known that one has to consider the load application conditions (e.g. the load type, application rate and angle), sample size and shape.

In the last years there is a large interest in TiB_2_ ceramic and composites reinforced with e.g. B_4_C and SiC^9–15^. These ceramics are investigated by bending tests usually at room temperature. In general, depending on grain size, reinforcement, and microstructure of the composite considering also defects, the room temperature bending strength attains values of 600–900 MPa. Macroscopic fracture mechanisms are related to cracks formation and development, these mechanisms being typical for brittle ceramics. Among them, literature indicates interfacial microcrack toughening due to thermal expansion coefficient differences of the composite components, cracks deflection, cleavage and enhancement of the intergranular fracture^14^. A much lower number of studies on the mechanical properties of these materials at high temperatures have been reported. In ref.^9^ are reviewed works presenting bending strength at high temperatures of TiB_2_. We learn that bending strength values also do not exceed 1GPa although a rising trend with an increasing temperature of the test is notable and deserves attention. It was recently reported that bending strength of TiB_2_-B_4_C composite achieves ultrahigh values up to 8.4 GPa at 2000 °C^16^. These values significantly exceed the limit of 1GPa for bending strength at room temperature.

Another ceramic of much interest is boron carbide (BC). Macroscopic fracture mechanisms at room temperature share similarities with those of TiB_2_, although crystal structure and chemistry are very different. The room temperature bending strength of BC-based ceramic composites also does not exceed 1GPa^17–19^. Bending strength of BC-based composites with temperature was reviewed in ref.^19^. The curves of bending strength versus temperature show a complex behavior and there are situations when, at high temperatures, bending strength exceeds the values measured at room temperature.

Previous two paragraphs suggest that mechanical properties studies of TiB_2_ and BC and of their composites at high temperatures can be rewarding. Expectations are that novel deformation mechanisms are active at high temperatures and they do not necessarily follow the conventional trends and understanding established for mechanical tests at room temperature. For example, few works^20–22^ reported ceramic composites of TiB_2_-BC, TiB_2_-TaC, or BC-TaB2 with a plastic behavior during bending at high temperatures, while an increased bending strength was also recorded. The unexpected increase in strength at high temperatures when compared to values at room temperature was linked with nano-twinning^23^, or twins’ rearrangement at high temperatures^19^. It is worthy to note, that while some mechanisms have been revealed in detail^24^, they are valid for very particular systems/classes of systems, thus not allowing to comprehend the full picture of the observed mechanical properties.

This paper reports bending strength exceeding 1 GPa at 1800 °C for the TiB_2_-BC composite. The curve of bending strength versus temperature presents a peculiar shape. The as-achieved high strength is partially explained by rearrangement of the crystallographic defects, such as twins and stacking faults toward a new system of nano-twins in the boron carbide grains. Details and a model of this mechanism are discussed. Previously, it was reported that in TiB_2_-BC composite, bending below 1600 °C shows an elastic-brittle behavior^20^, while at 2000 °C plastic deformation accompanied by amorphization of BC and strengthening are active^17^. As already mentioned, the bending strength at 1600 °C was below 1 GPa, while at 2000 °C it was much higher, in the range of 1.2–8.4 GPa. This result is not fully understood, it requires additional studies at intermediate bending temperature, and this is the aim of this work.

## Methods

**Figure 1:**
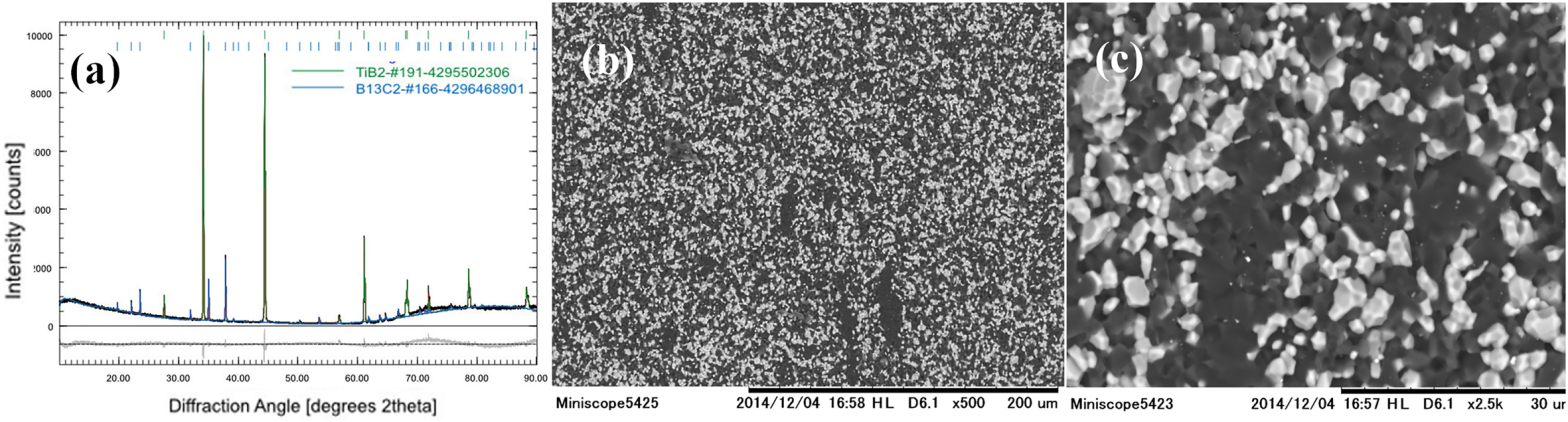
The TiB_2_-BC composite after bending at 1800 °C: (**a**) XRD pattern and Rietveld refinement; (**b**) and (**c**) SEM micrographs in backscattering regime at different magnifications (light grains are TiB_2_ and dark ones are BC).

Samples were prepared by spark plasma sintering using the ‘Dr. Sinter’ apparatus (Sumitomo, Japan) at 1900 °C for 10 min in Ar (2 L/min flow) from a mixture of TiB_2_ (Wako Pure Chemicals, Japan, purity 99%) and BC (International Labs, USA, purity 99%) powders (see Fig. 1 Supplementary material) with composition (TiB_2_/BC = 70/30 in vol%). Initially, a pressure of 20 MPa was applied to ensure sufficient electric contact between the powder mixture and the graphite die. Heating was with a rate of 100 °C min^−1^ up to 800 °C. After holding the sample at this temperature for 1 min, further heating was performed with a rate of 200 min^−1^. The uniaxial pressure was gradually increased during heating up to 60 MPa. Sample was cooled from 1900 to 1800 °C with a rate of 25 °C min^−1^ and to room temperature with a rate of 50 °C min^−1^. The pressure was decreased from 60 to 20 MPa between 1800 and 1700 °C. The pressure of 20 MPa was preserved until the end of the SPS consolidation.

The bulk density of the sample measured by Archimedes method was ~ 99% of the theoretical density evaluated by X-ray diffraction.

X-ray diffraction measurements were performed with a D8 Advance (Bruker, Karlsruhe, Germany) diffractometer (CuKα radiation). *Profex* software was used for Rietveld simulation.

The as-sintered bulk specimen of 30 mm in diameter was cut into bars (h × w × l = 2 mm × 2.3 mm × 20 mm) with a diamond disc and polished with the diamond paste down to 0.5 µm. Three-point bending strength was determined according to Japanese Standard JIS R160 (corresponding to ASTM C1211-13, configuration A) at 1800 °C in Ar atmosphere. Experiments were conducted with a Shimadzu AG-X plus system (Shimadzu, Japan)^22^. The span of 16 mm and loading speed of 0.5 mm/min were used. The heating schedule up to bending temperature was: from room temperature to 200 °C in 10 min and from 200 °C to the testing temperature at a rate of 18 °C·min^−1^. A dwell time of 5 min was employed before the flexural test at the testing temperature. After testing, cooling from the testing temperature to room temperature was performed at a rate of 20 °C·min^1^.

Microstructure was observed by scanning electron microscopy (SEM). The instrument is Tescan Lyra 3XMU and it is equipped with a focused ion beam facility. Details at high magnification were revealed by transmission electron microscopy (TEM) with a JEOL 2100 microscope. TEM investigations were performed on the sintered bar after bending. A thin sample (sample *E*) was obtained from the central region of the cross-section located at one of the free bar endings. Fabrication of this sample was by standard cross sectional (XTEM) method, involving ion milling (GATAN Precision Ion Polishing System). A second thin lamella (sample *F*) was extracted by FIB processing from an area in the vicinity of the fracture and in the middle of the bar. Under the assumption that the free end of the bar is subject to much lower internal stress than at the middle where the material is fracturing, the two analyzed sections are considered meaningful for a microstructural description of the bending process evolution.

## Results and discussion

Analysis of the XRD pattern from Fig. 1a indicate the presence of titanium boride TiB_2_ (CIF 4295502306) and boron-carbide B_13_C_2_ (CIF 4296468901) phases. The BC indicated stoichiometry was ascribed considering the best matching between our experimental XRD data and the available powder diffraction files. However, it is well known that BC is defined as a solid solution^25^ and precise determination of its stoichiometry is challenging^26^. Very recent advances^27^ propose more reliable, improved methodologies in this regard by using TEM facilities, but this is not straightforward, yet, and further refinements are still needed. Scanning Electron Microscopy (Fig. 1b,c) images taken in backscattering regime show close-packed faceted grains with a significant Z-contrast difference.

**Figure 2:**
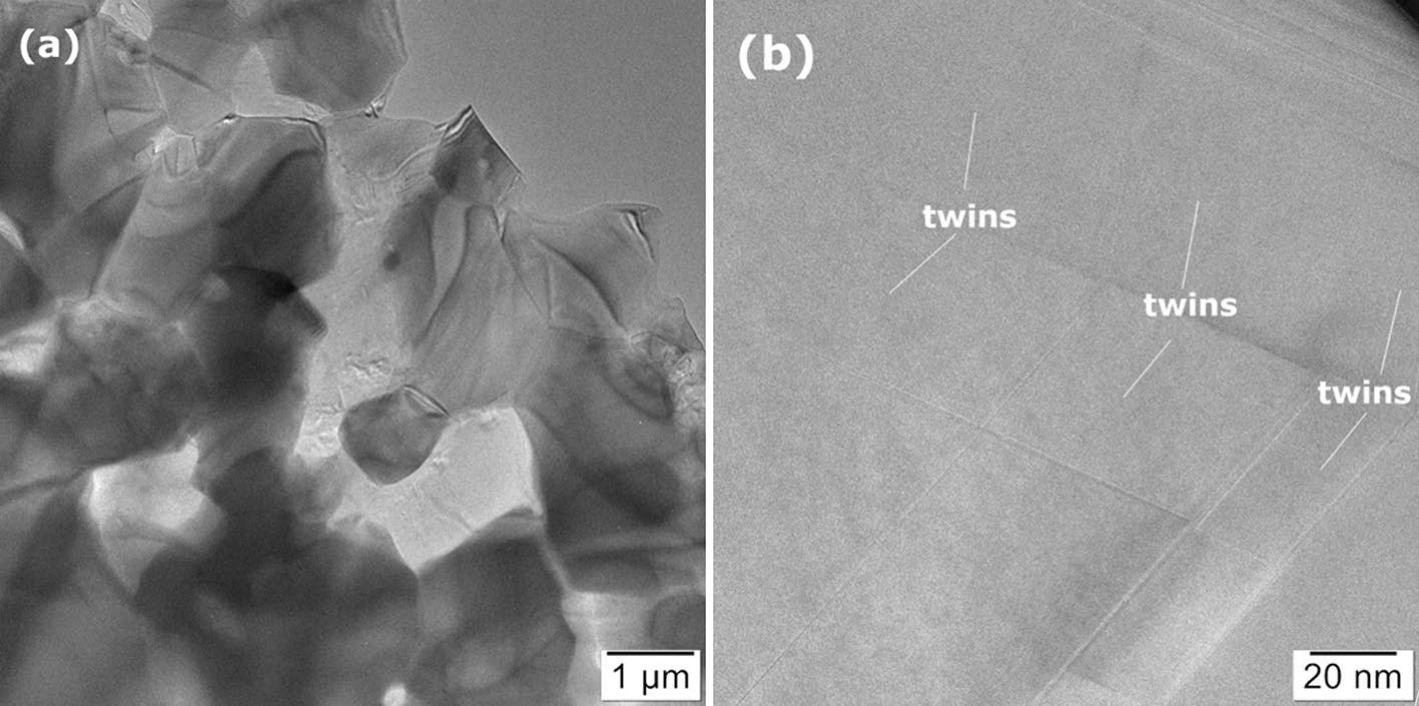
Sample E from the undeformed end of the TiB_2_-BC bar after bending test at 1800 °C (**a**) Low magnification BF-CTEM image showing TiB_2_ (darker) and BC (brighter) grains; (**b**) HRTEM image showing the system of stacking faults and twins in the BC grains.

The darker grains correspond to a low effective atomic number, namely BC, whereas whiter grains with a higher effective atomic number are of TiB_2_. Considering the definitions and classification of morphologies in composites from ref.^28^, results suggest that the material is composed of two interpenetrated 3D networks formed by TiB_2_ and BC grains. At least the BC network appears to be continuous. Information obtained by SEM is supported by TEM observations. Namely, in Fig. 2 conventional TEM (CTEM) images of TiB_2_ and BC can be visualized. The CTEM images were obtained in bright field (BF) mode, at the lowest magnification compatible with the electron-transparent area of the sample. Due to a strong contribution of the mass-thickness contrast, the darker grains have been associated with TiB_2_, whereas the brighter ones with BC. The CTEM image indicates that the crystal grains are roughly 1–2 µm. In Fig. 2b a high-resolution detail of BC grain with stacking faults and twins is shown. Additional details and differences between the samples *E* and *F* extracted after bending at 1800 °C from the free end and from a location close to the fractured area (see “Methods” section) were revealed. They are addressed in the next paragraphs.

**Figure 3:**
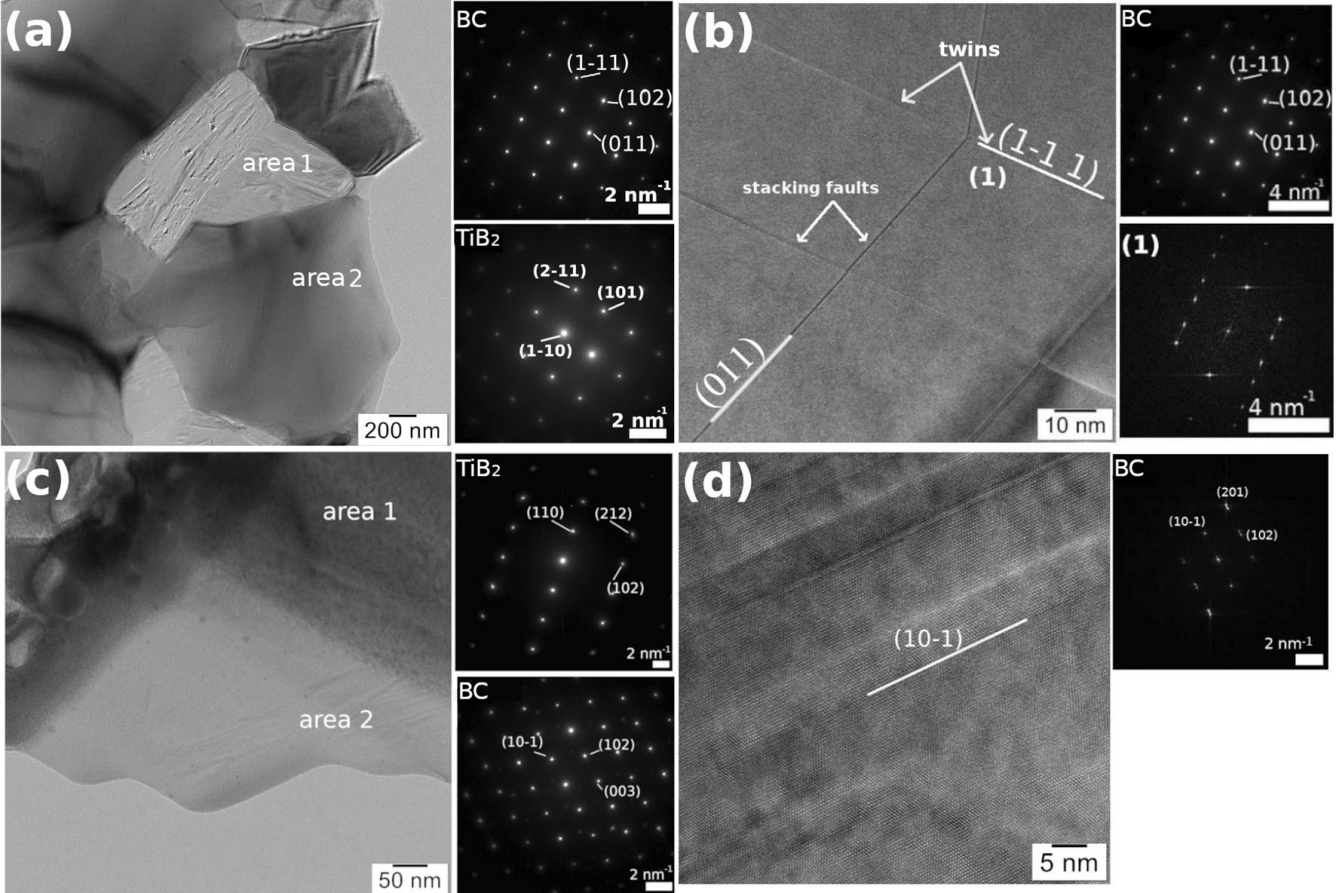
Sample E from the free end of the TiB_2_-BC bar after bending test at 1800 °C; (**a**) CTEM and selected area electron diffraction (SAED) images of area 1 = BC and area 2 = TiB_2_; (**b**) HRTEM and SAED images showing stacking faults and twins (1) in the BC grain. Sample F near the fracture in the TiB_2_-BC bar after bending test at 1800 °C; (**c**) CTEM and SAED images where area 1 = TiB_2_ and area 2 = BC crystal grains; (**d**) HRTEM and SAED images showing nano-twins in BC.

For sample *E* extracted after bending from the undeformed end of the bar, on the grains presented in the CTEM image from Fig. 3a, selected area electron diffraction (SAED) confirms the statement from the previous paragraph on the presence of the TiB_2_ and BC phases in Fig. 2a. High resolution image (HRTEM) of stacking faults and twins within the BC grains is shown in Fig. 3b. The twinning plane is (1-11) and the faults occur in the (1-11) and (011) plane stacks. We note that in the hexagonal family system of the BC crystal, the (011) and (1-11) are equivalent planes within the orthonormal basis of the crystals reciprocal space.

In sample *F* cut from the fracture area of the sintered bar after bending at 1800 °C, CTEM in the electron-transparent part shows the interface between two grains (Fig. 3c). SAED indicates that the two observed grains are TiB_2_ and BC. The TiB_2_ (space group 191, a = b = 3.02920 Å, c = 3.22 Å) and BC (space group 166, a = b = 5.653 Å, c = 12.156 Å) are both part of hexagonal crystal family. High resolution imaging of BC (Fig. 3d) indicates on a complex system of nano-twins (width < 5 nm) with (10-1) twinning planes. This plane is equivalent to the above mentioned (011) and (1-11) planes found in sample *E*.

**Figure 4:**
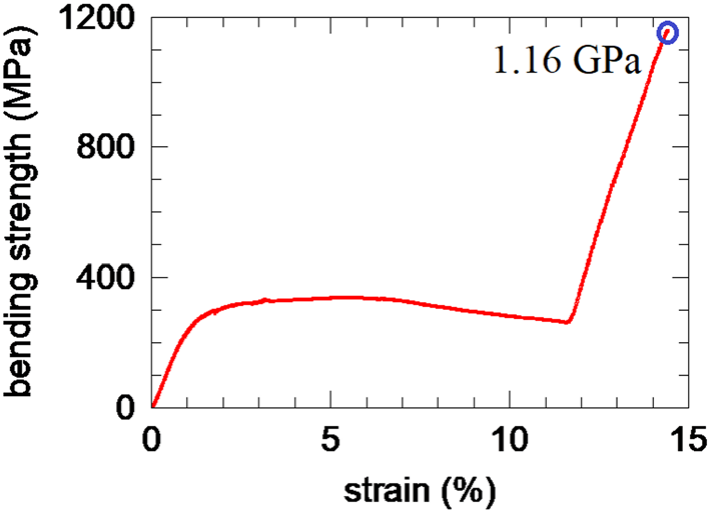
Typical bending strength curve at 1800 °C measured on the TiB_2_-BC sample obtained by SPS.

Stacking faults in sample *E* have been analyzed using the *Strain*++ software^29^. The reference coordinates system for strain computation was aligned with Oy axis along (1-11) crystallographic planes of BC. The tensile/compressive strain along Ox and Oy axes and the shear strain have been analyzed in the vicinity of 2 stacking faults (Fig. 4b). The Eps_xx_ and Eps_yy_ denote the tensile/compressive strain along Ox and respectively Oy, while the Epsxy and Epsyx are the labels for the shear strain (due to strain tensor symmetry they should have similar values). The computed values are relatively high for a local strain and suggest a significant shear strain in the stacking faults. Similarly, high values for a local stress have been observed in other situations^30,31^.

A typical strength-strain curve measured at 1800 °C is presented in Fig. 4. The curve is complex. There is an elastic region, followed by a plastic deformation for a strength around 300 MPa (where strength is constant or slightly decreases), and a strengthening region relatively linear, extending up to a bending strength over 1 GPa. The measured bending strength values at 1800 °C on 4 samples were between 1.08 and 1.7 GPa.

Complex non-linear profiles of the bending curves are found in literature. High similarities (i.e. the elastic, plastic and strengthening regions of deformation are distinguished) are with compression curves at room temperature measured on elasto-plastic structural polymeric composite foams^32^ and on porous ancient roman bricks^33^. Tensile tests also show a plastic region in the case of structural foams^32,34^. Elastic and plastic regions in room temperature bending tests were reported for fiber reinforced concrete^35,36^. Microcompression tests reported in ref.^37^ demonstrated room temperature plasticity in flash-sintered TiO_2_ oxide ceramic. The effect was attributed to the formation of nanoscale stacking faults and nano twins, which may be assisted by the high-density preexisting defects and oxygen vacancies introduced by the flash-sintering process. As already mentioned in section “Introduction”, boride and carbide ceramics may also exhibit high ductility at high temperatures resembling the plastic behavior of metals^5,6,8^. Extremely large deformation strains up to 50% were reported in ref^8^. for bending at 2000 °C of HfB_2_ ceramic. Within the temperature range from 900 to 1500 °C, a work hardening effect was also observed. No evidence of twinning was found and dislocation plasticity (pure dislocation glide without climb) by classical cold deformation model for metals was inferred. The composite investigated in this work contains TiB_2_ that shares the same crystal structure (Hermann-Mauguin space group P6/mmm) with ZrB_2_ and HfB_2_. However, evidence of high temperature plasticity, up to 1600–2000 °C in both TiB_2_^9,38^ and ZrB_2_^39,40^ was not reported. Lack of defects in the TiB_2_ grains from our TiB_2_-BC ceramic after high temperature bending, as indicated by the above presented TEM results, and the specific composite morphology with a 3D continuous network of BC suggest that the main contribution in defining the strength-strain curve profile is from BC. Not only the status of the defects in the two phases is different, but we shall also note that the crystal chemistry is different as reflected e.g. by different melting temperatures of 2763 °C and 3230 °C of B_4_C and TiB_2_, respectively. Therefore, this difference suggests that a higher possibility of strain activated defect-based processes under mechanical load at bending temperature of 1800 °C would be for the phase with a lower melting temperature, i.e. for BC. To get the extended picture of the mechanical response of the TiB_2_-BC composite, we shall also define the context of this experiment by providing a brief summary on our previous results. Remarkably, TiB_2_-BC has an ultrahigh bending strength of up to 8.4 GPa at 2000 °C, in inert Ar atmosphere. Mechanical response of the material shows at this temperature a significant plastic deformation (with strains above 10%), while also a process of strengthening occurs. After bending, authors observed by TEM large amorphous regions that can be responsible for the ductile behavior. Below 1600 °C^20^ the composite shows a typical brittle response without a plastic behavior and the fracture strength does not exceed 1GPa. These comparative details, as well as the fact that amorphous regions were not present in the samples tested at 1800 °C, strongly suggest that at intermediate bending temperatures new deformation situations occur and they are of much interest both from fundamental and practical viewpoints. Formation of cavities during bending above 1600 °C around the TiB_2_ grains with deformation of B_4_C grains was proposed in ref.^20^, but their number was quite low to solely explain the observed behavior, especially the occurrence of significant plasticity (strains over 10%). Cavities formation evidence the contribution of TiB_2_ grains in the composite as they develop at TiB_2_-BC grain boundaries.

**Figure 5:**
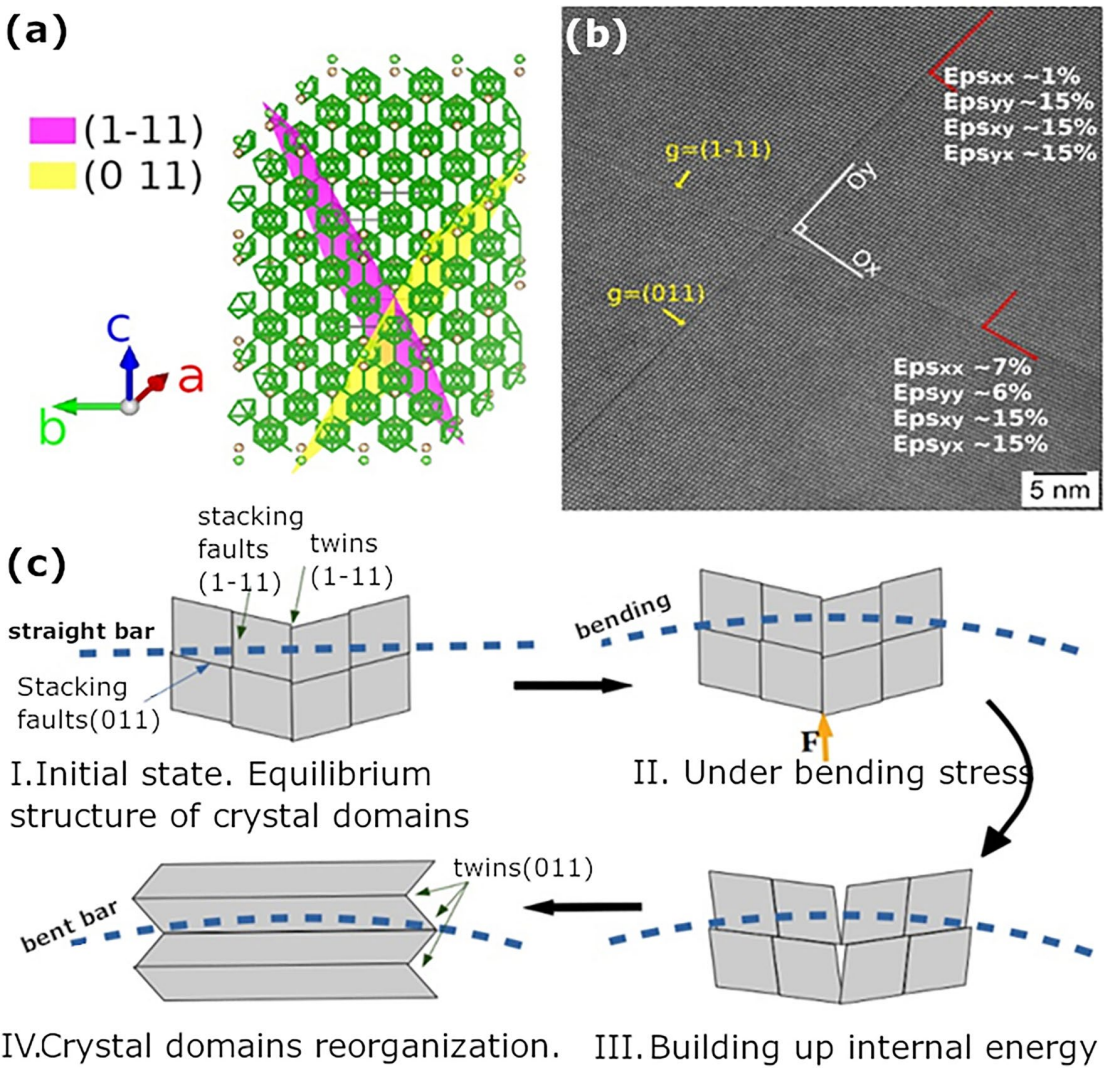
(**a**) Boron carbide crystal representation by VESTA software showing equivalent planes (1-11) and (011); (**b**) HRTEM image taken on sample E showing strain components with Oy being parallel to (011) planes; (**c**) Schematics of the transition under bending load between a twinned and stacking faulted structure as in sample E to a nano-twinned one as in sample F.

Based on addressed results and presented information from literature, the reorganization of the initial system of stacking faults and twins from the BC grains as in the sample *E* into the new twin system as in sample *F* under bending stress is inferred. It is proposed that the reorganization of the indicated defects takes place through the following mechanism (Fig. 5c): An external force (during bending process) applied along the (1-11) twinning planes (stage I) may force the twinned domains to rotate and align (stage II). As the deformation on one direction (i.e. along the twinning planes) is constrained by the external applied force (but it is also influenced by BC-BC or TiB_2_-BC grain boundaries and defects in BC that may oppose sliding), due to energy dissipation reasons, a shearing on orthogonal direction starts to take place (stage III), acting especially along the (011) stacking faults which are more susceptible to crystallographic rearrangement. The system reaches an energetically stable state when the shearing provides enough energy for the crystal structure to be rearranged into nano twins (stage IV). The model is a local one and, once the entire real randomly oriented polycrystalline material is considered, stages I-IV will occur and develop simultaneously due to the local distribution of the strain. Thus, under the increasing load that is enhancing the measured bending strain, the observed overall material’s response (see the bending strength-strain curve from Fig. 4) will be determined by the local processes from stages I–IV cumulated over the entire sample and by the interplay of their weight.

Based on the proposed model, the interpretation of the bending strength-strain curve from Fig. 4 is the following:

Under bending load at high temperature (1800 °C), after elastic deformation, stacking faults are prone to sliding providing plastic deformation and multiplying the number of (1-11) twins (stages I and II). The processes related to stacking faults and twins try to adapt the system to the energy injected by the bending load and their contribution in energy absorption is different. Shear strain can increase and promote formation of new twins with a twin plane rotated orthogonal (within the orthonormal base of the reciprocal space) to the plane of the initial stacking faults and twins (stage III). Some balance is achieved overall, at macro level, and the material sustains deformation so that the bending strength is approximately constant with strain in apparently a plastic-deformation-type behavior (Fig. 4, approximately horizontal region of the strength-strain curve).

When the process of twin rearrangement is almost completed, the new twins in the most stable configuration (stage IV) will play a significant role in material’s strengthening, pushing the bending strength to high values within a deformation behavior that becomes elastic-like at high strain values. The ultimate fracture strength exceeds 1 GPa. This is a relatively high value suggesting that the proposed mechanism can be also active and contribute to the observed strengthening in the TiB_2_-BC samples bent at 2000 °C, as described in ref.^16^.

An interesting observation is that in some cases, shear-induced phase boundaries were reported to produce in boron carbide materials asymmetric twins^7,19^. Twins rearrangement acknowledging the important role of stacking faults has been in-situ observed by TEM in Mg samples^24^. However, metals and ceramics are different although at high temperatures deformation in ceramics may share some similarities with room temperature deformation of metals^8^, as already pointed out. High strength measured on BC in ref.^23^ was ascribed to nano-twins with sizes similar to those from this work where the twin boundary slip is suppressed. For the composite from this work, a rearrangement switch of the twin boundary on the orthogonal direction can block the twin boundary slip and contribute strengthening.

In summary, the proposed twins’ rearrangement mechanism can explain the peculiar shape of the strength-strain curve, but other strengthening and fracturing mechanisms can also contribute. The role of TiB_2_ in the composite should not be neglected. We have not observed specific defects in TiB_2_ such as dislocations. In TiB_2_ single crystals, a plastic flow associated with the formation and dynamics of crystallographic defects was not observed at 1500 °C and below this temperature^38^. On the other hand, it was reported in ref.^41^ that in ZrB_2_ with a similar to TiB_2_ crystal structure and a brittle behavior under mechanical load and at high temperatures, dislocations occur. However their rearrangement is fast and they can easily vanish once the material fractures and the load is not active anymore at elevated temperatures.

Bending strength in air of B_4_C-ZrB_2_ composite show an elastic behavior (linear) up to 1600 °C temperature^40^, but when temperature increases the bending strength decreases, while the failure strain increases. We remind that in this work bending was performed in an Ar inert atmosphere. However, some contamination with oxygen is possible. Also some intake of carbon from the graphite die system used in the SPS processing might occur and contribute the mechanical properties. For bending in Ar, TiB_2_ remains essentially elastic, but according to ref.^42^ up to temperatures of ~ 1000 °C the elastic modulus slightly decreases. Above 1200 °C it has an accelerated decrease with temperature^8,9^. Changes in elasticity may influence local strain distribution, energy absorption and, thus, the response of BC from the TiB_2_-BC composite to load at different temperatures.

Abzianidze et al.^43^ observed in boron carbide ceramic an enhancement of the bending strength above 1000 °C and it was related to the development of the micro-ductility towards a maximum at the temperature of the brittle-ductile transition. This was considered the result of thermally-activated relaxation of the local peak stresses near the structural defects. A further increase in temperature (> 1600 °C) causes development of the macro-ductility and a monotonic decrease in strength occurs. Micro and macro ductility mechanisms were not discussed. Boron carbide has also another peculiar feature. Namely, its volume thermal expansion coefficient is not linear versus temperature. It increases up to a maximum value at about 1000 °C^44^ and it shows another smaller peak at around 700 °C.

The last three paragraphs indicate that information on mechanical behavior at high temperatures of both BC and TiB_2_ need clarifications. Further research is also required as not only the mechanical properties of TiB_2_ and BC, but also their complex interaction within the composite system should be evaluated for a complete understanding.

## Conclusions

Results indicate that under bending load at high temperatures boron carbide in the studied TiB_2_-BC composite presents an energetically favorable rearrangement of the stacking faults and twins. This is largely due to the equivalence of the crystalline planes (1-11), (011), and (10-1) of boron carbide within the hexagonal crystal family and due to the relatively significant shear strain component. The proposed mechanism of defects rearrangement, provides plasticity to the system followed by elastic-like strengthening. Shear strain component leads to a rearrangement of the twin boundary to an orthogonal crystallographic direction. This process, in a first instance, accommodates the energy introduced in the material within a plastic deformation behavior, and, afterwards, blocks the twin boundary slip promoting strengthening. It partially explains the peculiar shape of the stress–strain bending curve at 1800 °C composed of a sequence of three regions for increasing deformation: (i) conventional increase of strength in an elastic manner; (ii) constant strength for a plastic behavior; (iii) elastic-like increase of strength until fracturing. Apart from the occurrence of ductility in this ceramic composite the main consequence is the strengthening of the composite, thus exceeding at 1800 °C a bending strength of 1 GPa.

## Supplementary Information

Supplementary Figure 1.

## Data Availability

The datasets generated during and/or analyzed during the current study are available from the corresponding authors on reasonable request.
