# Supplementary material for: High bending strength at 1800 °C exceeding 1 GPa in TiB2-B4C composite
Source: Sci Rep. 2023 Apr 27;13:6915. doi: 10.1038/s41598-023-33135-w (PMC10140271; doi:10.1038/s41598-023-33135-w)
Supplement: Supplementary file 1 — Supplementary Figure 1. [file 41598_2023_33135_MOESM1_ESM.docx]

High bending strength at 1800 °C exceeding 1GPa in TiB_2_-B_4_C composite

A. Kuncser^a^, O. Vasylkiv^b*^, H. Borodianska^b^, D. Demirskyi^c,d*^, P. Badica^a*^

^a^National Institute of Materials Physics, street Atomistilor 405 A, 077125 Magurele, Romania

^b^National Institute for Materials Science, 1-2-1 Sengen, Tsukuba, Ibaraki 305-0047, Japan

^c^WPI-Advanced Institute for Materials Research (WPI-AIMR), Tohoku University, 2-1-1 Katahira, Aoba-ku, Sendai, 980-8577 Japan

^d^Department of Materials Science and Engineering, Tohoku University, 6-6-02 Aramaki Aza Aoba, Sendai, 980-8579, Japan

**Supplementary information**


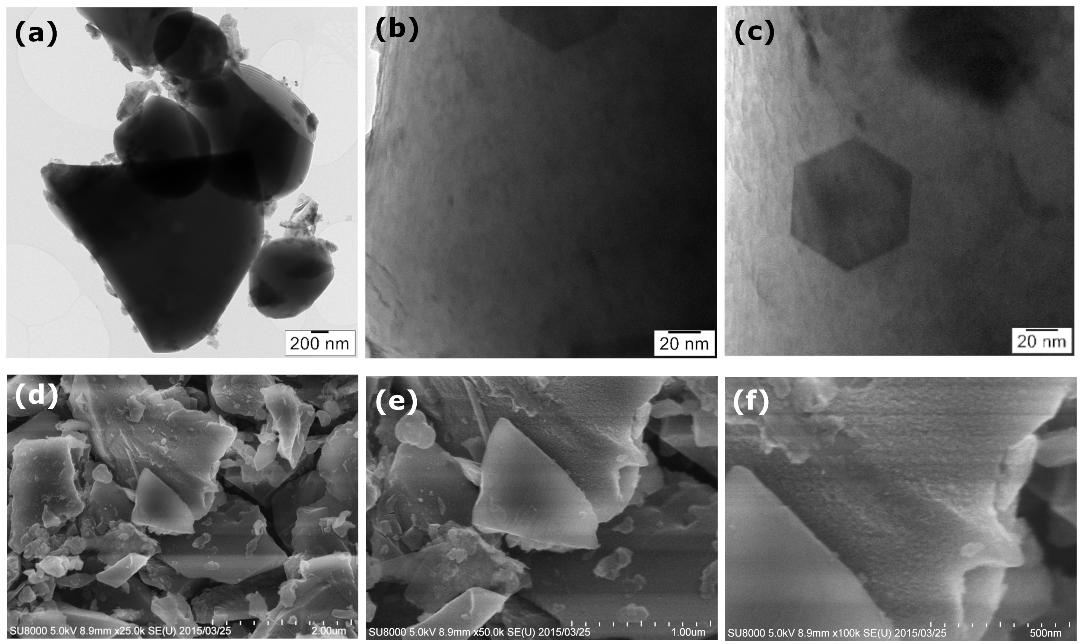


**Figure 1, Supplementary.** TEM and SEM images taken on raw (a)-(c) TiB_2_ (purity 99 wt. %) and (d)-(f) BC (purity 99 wt. %) powders. The average particle size of TiB_2_ is 1.8 µm (impurities are 0.8 wt. % oxygen, 0.08 wt. % carbon and 0.12 wt. % nitrogen), while for BC it is 1.5 µm (impurities are 0.08 wt. % oxygen and 0.02 wt. % Si and metals).
